# Early Changes in Volumetric Body Composition Parameters Predict Survival Outcomes in Patients with Metastatic Renal Cell Carcinoma Treated with Targeted Therapy

**DOI:** 10.3390/jcm14093140

**Published:** 2025-05-01

**Authors:** Oktay Halit Aktepe, Ahmet Gurkan Erdemir, Eda Caliskan Yildirim, Erkut Demirciler, Tugce Ulasli, Deniz Can Guven, Mehmet Ruhi Onur, Ilkay Tugba Unek, Huseyin Salih Semiz, Mustafa Erman, Suayib Yalcin

**Affiliations:** 1 Department of Medical Oncology, Dokuz Eylul University, 35330 Izmir, Turkey; tugce.ulaslialtun@deu.edu.tr (T.U.); ilkaytugbaunek@gmail.com (I.T.U.); hsalihsemiz@hotmail.com (H.S.S.); 2 Department of Radiology, Faculty of Medicine, Hacettepe University, 06230 Ankara, Turkey; ahmet.erdemir@hacettepe.edu.tr (A.G.E.); ruhionur@hacettepe.edu.tr (M.R.O.); 3 Department of Medical Oncology, Sincan Research and Training Hospital, 06949 Ankara, Turkey; caliskan_eda@yahoo.com; 4 Department of Medical Oncology, Izmir City Hospital, 35540 Izmir, Turkey; erkutdemirciler@gmail.com; 5 Department of Medical Oncology, Hacettepe University Cancer Institute, 06230 Ankara, Turkey; guvendenizc@gmail.com (D.C.G.); ermanm1968@gmail.com (M.E.); suayibyalcin@gmail.com (S.Y.)

**Keywords:** volumetric body composition, metastatic renal cell carcinoma, skeletal muscle index, prognosis

## Abstract

**Background/Objectives**: The precise role of volumetric body composition (VBC) parameters, visceral adipose tissue index (VATI), subcutaneous adipose tissue index (SATI), and skeletal muscle index (SMI) on the survival of metastatic renal cell carcinoma (mRCC) is not fully elucidated. Herein, the present study investigated the clinical significance of baseline VBC parameters and their changes after 3–4 months from treatment initiation in patients with mRCC treated with first-line targeted therapy. **Methods**: A total of 108 patients were enrolled. VBC parameters were depicted from computerized tomography (CT) images at the third lumbar vertebra level. Kaplan–Meier curves were used to estimate survival probability, and the differences between prognostic subgroups were compared with the log-rank test. The association of baseline VBC variables and their change values (First CT value minus baseline CT value) with progression-free survival (PFS) and overall survival (OS) was evaluated in univariate and multivariate analyses. **Results**: The median PFS and OS of the whole patients were 11 and 46 months, respectively. Patients with increased VATI and SATI change values had poorer OS than those with decreased values. However, patients with higher SMI change values had superior OS than those with lower values. Among VBC variables, the independent predictors of worse OS were high VATI change (HR 5.10, *p* = 0.001) and low SMI change values (HR 2.66, *p* = 0.007), in addition to International Metastatic Renal Cell Carcinoma Database Consortium prognostic stratification (*p* = 0.001). **Conclusions**: Our findings showed that high VATI and low SMI changes were associated with worse OS in mRCC patients treated with first-line targeted therapy.

## 1. Introduction

Globally, renal cell carcinoma (RCC) ranks as the sixth most commonly diagnosed cancer in men and the tenth in women, accounting for 4% of the overall cancer burden [1]. Although most lesions detected incidentally through advanced imaging techniques are small and low-grade tumors, 17% of all RCC cases present with distant metastasis at diagnosis [2]. Thanks to groundbreaking advancements in recent years, the first-line treatment for metastatic RCC (mRCC) has evolved from cytokine-based therapies to include inhibitors of the vascular endothelial growth factor (VEGF) pathway, immune checkpoint inhibitors (ICIs), and their combinations [3]. To determine the prognosis for mRCC patients, the International Metastatic Renal Cell Carcinoma Database Consortium (IMDC) developed a prognostic scoring system, classifying mRCC patients into risk levels: favorable, intermediate, and poor [4]. Even though obesity is linked to an increased risk of RCC, the data regarding the mechanisms by which obesity may heighten this specific cancer’s risk is insufficient [5]. On the other hand, hypotheses suggest that the biological mechanisms through which obesity contributes to RCC may include chronic tissue hypoxia, alterations in the hormonal environment of fat tissue, and immune dysfunction [6,7]. Interestingly, some studies indicate that obese mRCC patients respond better to treatment and have a more favorable prognosis; this phenomenon is referred to as the ‘obesity paradox’ in the literature [8,9]. This ‘risk factor’ paradox may arise from the temporal gap between two harmful risk factors: overnutrition, a well-known long-term risk, may act as a protective factor in the short term, while malnutrition poses a short-term risk [10]. Additionally, the decline in skeletal muscle, known as sarcopenia, has been identified as a predictor of prognosis in various tumors, including RCC [11,12,13]. Furthermore, it has been shown that the reduction in skeletal muscle mass correlates with increased side effects from anti-cancer treatments [14] and poorer overall survival (OS) rates in sarcopenic obese patients [15]. Body mass index (BMI) is a rough measure of fat distribution in the body, making it insufficient alone to ascertain the exact body composition of sarcopenia, characterized by the loss of body fat and skeletal muscle tissue. Computerized tomography (CT) is routinely used to assess the staging and treatment response of RCC, enabling better categorization of fat and skeletal muscle tissue. Additionally, CT can classify fat tissue relative to its visceral and subcutaneous compartments [visceral fat area (VFA) and subcutaneous fat area (SFA)]. Studies investigating the effects of these parameters on the prognosis of mRCC have shown varying results. Steffens et al. [9] and Gu et al. [16] demonstrated that higher VFA and SFA in mRCC patients correlate with prolonged OS. In contrast, increased VFA was associated with poor outcomes in Ladoire’s cohort [17]. Antoun et al. found no correlation between VFA or SFA and survival outcomes in patients with mRCC [18]. A cross-sectional observational study has shown that body weight and tissue loss can contribute to predicting the prognosis of mRCC patients treated with targeted therapies [19]. However, the clinical significance of longitudinal changes in volumetric body composition (VBC) parameters over time in mRCC patients treated with targeted therapy remains unclear. The current study aimed to investigate whether obesity and VBC parameters measured in terms of visceral adipose tissue index (VATI), subcutaneous adipose tissue index (SATI), skeletal muscle index (SMI), and their changes after 3–4 months following the initial CT can predict survival outcomes in patients undergoing targeted therapy.

## 2. Materials and Methods

The study population consisted of 108 Caucasian patients with confirmed mRCC, all aged 18 years or older, who received anti-angiogenic therapy from January 2008 to May 2019 at Hacettepe University Cancer Institute in Ankara, Turkey. Key demographic and oncologic characteristics of the patients—including age, gender, IMDC risk score, number of metastatic sites, treatment records, tumor grade, and histologic subtype—were collected from our center’s electronic recording system. Only patients with histologically confirmed mRCC who underwent first-line targeted therapy were included. BMI was calculated as weight (kg) divided by height (m^2^). All patients had baseline and subsequent contrast-enhanced CT images taken 3–4 months after the initiation of targeted therapy to evaluate treatment response. Patients lost to follow-up but possessing an analyzable CT scan were excluded from the study. All procedures were conducted according to the ethical standards of the institutional and/or national research committee and the 1964 Helsinki Declaration, along with its later amendments. The local ethics committee approved the study, and written informed consent was waived due to the retrospective archival nature of the study.

### 2.1. Imaging Analysis

**Figure 1:**
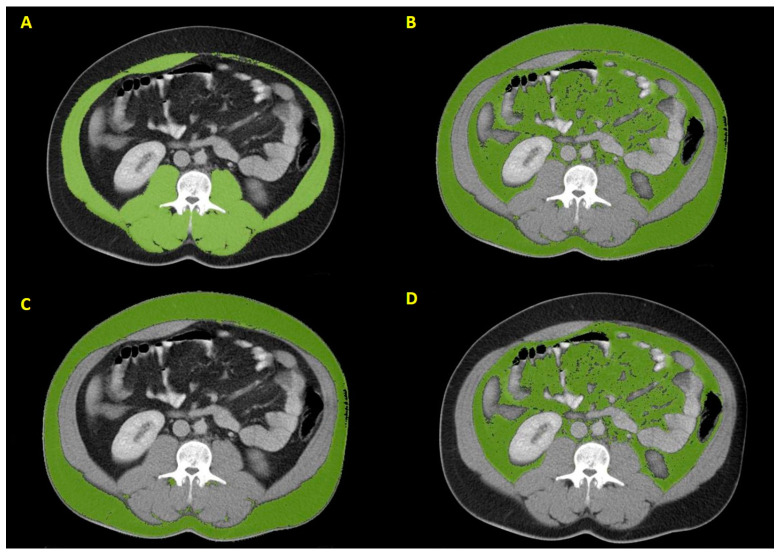
Axial CT images at the level of the L3 vertebra illustrate computer-assisted tissue segmentation for analyzing VBC. The green-highlighted regions show SMA (**A**), total adipose tissue area (**B**), SFA (**C**), and VFA (**D**).

Radiological measurements were conducted by two radiologists in collaboration (M.R.O., A.G.E.) who were blinded to all patient clinical information. Contrast-enhanced CT images of all patients, with a section thickness of 5.0 mm at the venous phase, were selected. For each CT image interpretation, axial CT images oriented parallel to the superior and inferior end plates of the L3 vertebra were chosen. The CT images were evaluated using the Region Growing application of Syngo.Via Software (Version VB40B, Siemens Healthcare GmbH, Erlangen, Germany). Within each cross-sectional CT image, SFA, VFA, and skeletal muscle area (SMA) were determined separately and outlined semi-automatically (Figure 1). These parameters were standardized according to stature and then named VATI, SATI, and SMI, represented in units of cm^2^/m^2^, as described previously [19]. The delta (Δ) value was obtained by subtracting the baseline measurements from the post-treatment measurements.

### 2.2. Statistical Analysis

Descriptive analyses are presented as medians and interquartile ranges for numerical data and as frequencies and percentages for categorical data. Independent groups were compared using the Mann–Whitney U test for numerical variables and the chi-square test for categorical variables. OS is defined as the time from treatment initiation to the last follow-up or death, while progression-free survival (PFS) is defined as the time from treatment initiation to progression or death. The median follow-up time was calculated from diagnosis to the last follow-up or date of death. Survival comparisons were conducted using the Kaplan–Meier method and the log-rank test. The receiver operating characteristic (ROC) curves were utilized to find optimal cut-off values of VBC parameters in predicting OS. Variables with a *p*-value of less than 0.20 were considered candidate variables for multivariate analyses. SPSS 25 software (IBM Inc., Armonk, NY, USA) was utilized for the analyses in this study, with a *p*-value below 0.05 regarded as statistically significant.

## 3. Results

### 3.1. Baseline Patient Characteristics

The baseline patient and tumor characteristics of 108 mRCC patients are presented by gender in Table 1. The median age was 57. Clear cell histology (77.8%) was the most common subtype. Most patients had two or fewer metastatic sites (81.5%). The metastatic sites included the lung (75.9%), liver (24.1%), bone (23.1%), adrenal gland (13%), and brain (3.7%). Our cohort predominantly consisted of tumors graded I-II. According to the IMDC risk score, patients were categorized into three prognostic subgroups: favorable (21.3%), intermediate (50%), and poor (28.7%). The entire study population received targeted therapy (pazopanib, 59.3%; sunitinib, 40.7%).

Body composition parameters differed between men and women, as shown in Table 2. Twenty-eight patients (25.9%) were considered obese (BMI of ≥ 30 kg/m^2^). While women had higher median baseline and first SATI values than men, the median change in SATI for women was lower compared to that of men. However, the median baseline SMI was higher in men than in women (*p* = 0.004). There were no differences between the two groups in baseline VATI, first VATI, and VATI change values by gender.

### 3.2. The Effect of Body Composition Parameters on Survival Outcomes

During a median follow-up of 22 months (range: 2–102 months), 38 patients (35.2%) died. The median PFS and OS times for the entire cohort were 11 months [95% confidence interval (CI) 8–13.9] and 46 months (95% CI 42.8–49.1), respectively. Among obese (BMI ≥ 30 kg/m^2^) and non-obese (BMI < 30 kg/m^2^) patients, Kaplan–Meier analysis indicated a significant difference in PFS, with a median of 14 months for obese patients and 8 months for non-obese patients (*p* = 0.039). However, no significant difference in OS was observed, with a median of 46 months for obese patients and 47 months for non-obese patients (*p* = 0.921). The median PFS times for each IMDC risk group were 21 months (favorable-risk group; 95% CI 15.6–26.3), 12 months (intermediate-risk group; 95% CI 9.6–14.3), and 6 months (poor-risk group; 95% CI 4.8–7.1) (*p* < 0.001). The median OS times for patients in the favorable and intermediate-risk groups were 60 months (95% CI 40.4–79.5) and 33 months (95% CI 27.5–38.4), respectively. In contrast, the median OS was not reached in the poor-risk group, with a mean OS during ongoing follow-up of 28.2+ months (*p* < 0.001).

ROC analysis indicated that the optimal cut-off values for ΔSATI, ΔVATI, and ΔSMI in predicting OS were 2.37 cm^2^/m^2^ (sensitivity: 67%; specificity: 74%; AUC: 0.77, 95% CI 0.68–0.86, *p* < 0.001), 1.1 cm^2^/m^2^ (sensitivity: 81%; specificity: 80%; AUC: 0.75, 95% CI 0.65–0.86, *p* < 0.001), and −2.25 cm^2^/m^2^ (sensitivity: 65%; specificity: 64%; AUC: 0.67, 95% CI 0.57–0.78, *p* = 0.002), respectively.

**Figure 2:**
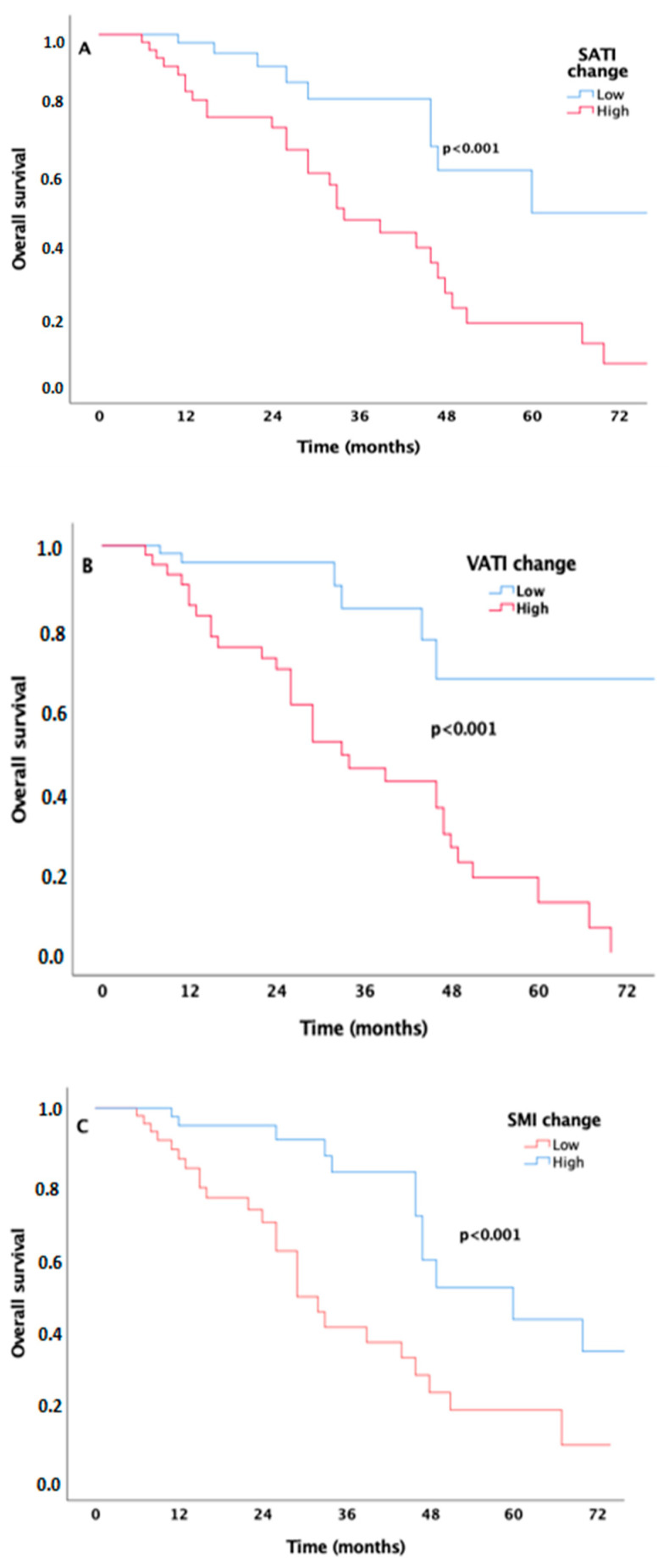
Kaplan–Meier curves estimate the survival probabilities of patients stratified by ΔSATI (**A**), ΔVATI (**B**), and ΔSMI (**C**).

Considering the clinical significance of the change values of VBC, patients with high ΔSATI (≥2.37 cm^2^/m^2^) and high ΔVATI (≥1.1 cm^2^/m^2^) exhibited shorter OS times compared to those with low values (34 months vs. 60 months, *p* < 0.001, Figure 2A; 33 months vs. 87 months, *p* < 0.001, Figure 2B). In contrast, the median OS of patients with high ΔSMI (≥−2.25 cm^2^/m^2^) was longer than that of those with low values (60 months vs. 29 months, *p* < 0.001, Figure 2C). Univariate analyses presented in Table 3 indicated that the variables associated with PFS included baseline CI SMI [Hazard ratio (HR): 0.97; 95% CI, 0.96–0.99, *p* = 0.011], ΔVATI (HR: 0.65; 95% CI, 0.43–0.99, *p* = 0.049), ΔSATI (HR: 0.61; 95% CI, 0.40–0.94, *p* = 0.028), and the IMDC score (*p* < 0.001). However, the variables identified for poorer OS were high ΔSATI (HR: 3.61; 95% CI, 1.70–7.66, *p* = 0.001), high ΔVATI (HR: 4.70; 95% CI, 1.82–12.15, *p* = 0.001), and low ΔSMI (HR: 3.29; 95% CI, 1.64–6.61, *p* = 0.001), in addition to the IMDC score (*p* = 0.001). Multivariate Cox regression models identified independent prognostic factors for PFS and OS, as shown in Table 4. The independent variables for PFS were the baseline SMI value (HR: 0.98; 95% CI, 0.96–0.99, *p* = 0.026) and the IMDC score (*p* < 0.001). The independent variables predicting poorer OS included high ΔVATI (HR: 5.10; 95% CI, 1.90–13.69, *p* = 0.001) and low ΔSMI (HR: 2.66; 95% CI, 1.30–5.45, *p* = 0.007), in addition to the IMDC score (*p* = 0.001).

## 4. Discussion

The development of VEGF pathway-blocking agents has resulted in a significant breakthrough in the outcomes of mRCC. Additionally, ICIs are used either alone [20] or in combination with VEGF-blocking agents to treat mRCC [21]. With numerous alternative therapies available, such as immunotherapy, novel prognostic markers are essential for providing enhanced personalized treatments. Current prognostic scoring systems have been established based on clinical and laboratory parameters. Although obesity is not among these criteria, its effect on mRCC prognosis remains unclear in the literature [22]. Therefore, in addition to BMI, this study examined the relationship between survival outcomes and VBC measurements, as well as their post-treatment changes from the third to fourth month in mRCC patients undergoing targeted therapy. The findings indicated that the independent predictive variables were the baseline SMI value for PFS, along with ΔVATI and ΔSMI for OS. To the best of our knowledge, this is the first analysis of the impact of early changes in VBC on the survival outcomes of mRCC patients.

Body composition is defined as the amount and distribution of lean tissue, fat tissue, and bone. Up to this decade, studies on body composition concerning clinical outcomes of mRCC have primarily relied on BMI. However, BMI cannot accurately distinguish between muscle and adipose tissue. With advances in imaging technologies such as CT, used for staging and response assessment, a more precise evaluation of muscle and fat is now possible. Therefore, in addition to BMI, studies have started measuring the SFA and VFA components of intra-abdominal fat tissue at the umbilicus level via CT. Steffens et al. investigated the impact of body surface area (BSA, m^2^), VFA, and SFA on the prognosis of 116 mRCC patients and demonstrated that contrary to BMI and BSA, above-average VFA and SFA are associated with longer PFS and OS in mRCC prognosis (SFA, HR: 3.41, 95% CI, 1.61–7.25, *p* = 0. 001; VFA, HR: 2.97, 95% CI, 1 36–6.47, *p* = 0. 006) [9]. In contrast to these findings, Ladoire et al. evaluated the prognostic value of BMI, SFA, and VFA in 64 French mRCC patients treated with targeted therapy, using a similar measurement technique as Steffens’ trial [9] and demonstrated that high VFA was significantly associated with worse OS (HR: 6.26, 95% CI, 2.29–17.08, *p* < 0.001) [17]. The conflicting results of these studies may be attributed to undefined cut-off points by gender and/or CT measurements evaluated at the umbilicus level, as it is inappropriate to use umbilicus-level CT measures to estimate total body fat and muscle tissue. Consequently, Antoun et al. reported no significant association between VFA and SFA with survival outcomes of mRCC when these measurements were performed at a more appropriate level, such as L 3, and adjusted for gender [19]. Recently, McManus et al. studied the relationship between baseline VBC and survival outcomes in mRCC patients treated with first-line nivolumab and ipilimumab and showed no significant association between VBC and OS [23]. Unlike that study, our study evaluated the effect of early changes (value at first CT minus value at baseline) of VBC on survival outcomes in mRCC patients treated with targeted therapy.

This study has several limitations. First, it was conducted at a single center with a relatively small sample size, which may limit the generalizability of the findings. Second, due to the unavailability of ICIs and their combination treatments with anti-VEGF agents as first-line options during the study period in our country, we were unable to assess the prognostic impact of VBC parameters in patients receiving these therapies. Third, although histological subtype may influence treatment outcomes, particularly in the context of immunotherapy, none of the patients in our cohort received such treatments, and clear cell RCC constituted the majority (~78%) of the cases. Therefore, a subgroup analysis based on histology would not have been statistically meaningful and could have introduced bias. Fourth, while sex-specific differences in VBC are acknowledged, the low number of female patients (n = 31) limited our ability to perform reliable sex-stratified analyses. Finally, since most patients were classified within the intermediate-risk group (50%), our findings should be further validated in larger and more homogeneous cohorts, especially including both favorable- and poor-risk groups.

## 5. Conclusions

This study indicated that early changes in VATI and SMI were independent predictors of OS in patients with mRCC who received systemic therapy. Additionally, baseline SMI was identified as an independent predictor for PFS. Considering the widely disparate results of the studies mentioned above, the prognostic value of VBC parameters and their changes during the course of mRCC should be evaluated in a larger patient population using a prospective design to validate the cut-off values for these parameters.

## Figures and Tables

**Table 1:** Baseline clinical–pathological characteristics stratified according to gender.

Characteristics	All Patients (n = 108)	Female (n = 31)	Male (n = 77)
Age [median (IQR)]	57 (51–64)	56 (50–64)	59 (52–64)
IMDC risk group [n (%)]			
Favorable	23 (21.3%)	6 (19.4%)	17 (22.1%)
İntermediate	54 (50%)	19 (61.2%)	35 (45.4%)
Poor	31 (28.7%)	6 (19.4%)	25 (32.5%)
Histology [n (%)]			
Clear cell	84 (77.8%)	27(87%)	57 (74%)
Non-clear cell	24 (22.2%)	4 (13%)	20 (26%)
Metastatic sites [n (%)]			
≤2	88 (81.5%)	24 (81.5%)	64 (81.5%)
>2	20 (18.5%)	7 (18.5%)	13 (18.5%)
Fuhrman grade [n (%)]			
I–II	88 (81.5%)	28 (90.3%)	60 (77.9%)
III–IV	20 (18.5%)	3 (9.7%)	17 (22.1%)
Treatment [n (%)]			
Pazopanib	64 (59.3%)	17 (54.8%)	47 (61%)
Sunitinib	44 (40.7%)	14 (45.2%)	30 (39%)

IQR: interquartile range, IMDC: International Metastatic Renal Cell Carcinoma Database Consortium.

**Table 2:** VBC parameters at the baseline and follow-up CT performed 3–4 months after treatment initiation.

Body Composition Variables	All Patients n = 108	Female n = 31 (28.7%)	Men n = 77 (71.3%)	*p*-Value
Height, cm	1.70 (1.64–1.75)	1.61 (1.58–1.65)	1.71 (1.69–1.75)	<0.001
Weight, kg	75 (69–82)	70 (63–77)	77 (70–83)	0.004
BMI (kg/m^2^)	25.9 (3.89)	26.7 (3.83)	25.8 (3.93)	0.541
Baseline VATI, cm^2^/m^2^	45.7 (32.6–72.5)	53.2 (33.4–80.1)	44.2 (30.7–66.9)	0.252
First VATI, cm^2^/m^2^	47.7 (30.2–71.5)	40.7 (30.6–75.8)	48 (30–67.9)	0.911
ΔVATI, cm^2^/m^2^	−2.3 (−8.1–6.2)	−3.5 (−8.8–3.6)	−1 (−7.7–7.2)	0.310
Baseline SATI, cm^2^/m^2^	54.5 (39.6–77.9)	76.2 (64.8–114.3)	47.5 (34–68.7)	<0.001
First SATI, cm^2^/m^2^	60.3 (39.7–77.6)	75.4 (60.5–112.6)	54.2 (31.6–71.5)	<0.001
ΔSATI, cm^2^/m^2^	−0.64 (−6.8–7.7)	−5.9 (−9.9–6.5)	2 (−5.13–7.8)	0.018
Baseline SMI, cm^2^/m^2^	49.2 (41.7–55.3)	43.5 (39.5–49.9)	51.8 (42.8–58.6)	0.004
First SMI, cm^2^/m^2^	46.9 (39.2–54.5)	44 (36.3–49.9)	47.5 (40.5–55.35)	0.082
ΔSMI, cm^2^/m^2^	−1.6 (−5.2–0.5)	−0.85 (−2.9–1.3)	−2.24 (−6.7–[−0.07])	0.067

BMI: body mass index; VATI: visceral adipose tissue index; SATI: subcutaneous adipose tissue index; SMI: skeletal muscle index.

**Table 3:** Univariate Cox regression models estimating the potential influence of clinical–pathological features and VBC parameters on PFS and OS.

	PFS		OS	
Characteristic	HR (95% CI)	*p*-Value	HR (95% CI)	*p*-Value
Age, years	1 (0.98–1.03)	0.464	1 (0.96–1.03)	0.937
Gender (Male vs. Female)	1.41 (0.91–2.18)	0.121	1.63 (0.74–3.58)	0.223
IMDC risk group		<0.001		0.001
Favorable	1 (reference)		1 (reference)	
İntermediate	1.66 (0.98–2.82)	0.06	2.59 (1.19–5.66)	0.016
Poor	6.95 (3.55–13.59)	<0.001	8.91 (2.95–26.92)	<0.001
Tumor grade (III–IV vs. I–II)	1.02 (0.63–1.65)	0.913	1.3 (0.63–2.69)	0.476
Histology (non-clear vs. clear)	0.85 (0.49–1.45)	0.553	1.33 (0.62–2.83)	0.453
Metastasis region site (III–IV vs. I–II)	1.09 (0.65–1.81)	0.737	0.43 (0.10–1.82)	0.256
BMI	0.95 (0.9–1)	0.223	0.94 (0.84–1.04)	0.245
Baseline VATI	0.99 (0.98–1)	0.251	0.99 (0.98–1)	0.185
ΔVATI (high value vs. low value)	0.65 (0.43–0.99)	0.049	4.7 (1.82–12.15)	0.001
Baseline SATI	1 (0.99–1)	0.945	0.99 (0.98–1)	0.168
ΔSATI (high value vs. low value)	0.61 (0.40–0.94)	0.028	3.61 (1.70–7.66)	0.001
Baseline SMI	0.97 (0.96–0.99)	0.011	1.01 (0.98–1.04)	0.345
ΔSMI (low value vs. high value)	1.5 (0.98–2.29)	0.056	3.29 (1.64–6.61)	0.001

IMDC: International Metastatic Renal Cell Carcinoma Database Consortium, BMI: body mass index, VATI: visceral adipose tissue index, SATI: subcutaneous adipose tissue index, SMI: skeletal muscle index.

**Table 4:** Multivariate Cox regression models analyze the potential influence of VBC parameters on PFS and OS.

	PFS		OS	
Characteristic	HR (95% CI)	*p*-Value	HR (95% CI)	*p*-Value
ΔSMI (low value vs. high value)			2.66 (1.30–5.45)	0.007
ΔVATI (high value vs. low value)			5.10 (1.90–13.69)	0.001
Baseline SMI	0.98 (0.96–0.99)	0.026		
IMDC risk group		<0.001		0.001
Favorable	1 (reference)		1 (reference)	
İntermediate	1.74 (1.02–2.97)	0.040	2.38 (1.06–5.34)	0.057
Poor	6.84 (3.48–13.47)	<0.001	9.58 (2.86–32.0)	<0.001

PFS: progression-free survival; SMI: skeletal muscle index; IMDC: International Metastatic Renal Cell Carcinoma Database Consortium; VATI: visceral adipose tissue index.

## Data Availability

The datasets used and/or analyzed during the current study are available from the corresponding author (Oktay Halit Aktepe, oktayhalit.aktepe@deu.edu.tr) upon reasonable request.

## Abbreviations

BSA	Body surface area
BMI	Body mass index
CI	Confidence interval
CT	Computerized tomography
HR	Hazard ratio
ICI	Immune checkpoint inhibitor
IMDC	International Metastatic Renal Cell Carcinoma Database Consortium
mRCC	Metastatic renal cell carcinoma
OS	Overall survival
PFS	Progression-free survival
RCC	Renal cell carcinoma
ROC	Receiver operating characteristic
SATI	Subcutaneous adipose tissue index
SFA	Subcutaneous fat area
SMI	Skeletal muscle index
VEGF	Vascular endothelial growth factor
VATI	Visceral adipose tissue index
VFA	Visceral fat area

## References

[1] Siegel R.L., Miller K.D., Jemal A. (2016). Cancer statistics, 2016. CA Cancer J. Clin..

[2] Capitanio U., Montorsi F. (2016). Renal cancer. Lancet.

[3] Ivanyi P., Fröhlich T., Grünwald V., Zschäbitz S., Bedke J., Doehn C. (2024). The Treatment of Metastatic Renal Cell Carcinoma. Dtsch. Arztebl. Int..

[4] Heng D.Y., Xie W., Regan M.M., Warren M.A., Golshayan A.R., Sahi C., Eigl B.J., Ruether J.D., Cheng T., North S. (2009). Prognostic factors for overall survival in patients with metastatic renal cell carcinoma treated with vascular endothelial growth factor–targeted agents: Results from a large, multicenter study. J. Clin. Oncol..

[5] Klinghoffer Z., Yang B., Kapoor A., Pinthus J.H. (2009). Obesity and renal cell carcinoma: Epidemiology, underlying mechanisms and management considerations. Expert Rev. Anticancer Ther..

[6] Calle E.E., Kaaks R. (2004). Overweight, obesity and cancer: Epidemiological evidence and proposed mechanisms. Nat. Rev. Cancer..

[7] Donat S.M., Salzhauer E.W., Mitra N., Yanke B.V., Snyder M.E., Russo P. (2006). Impact of Body Mass Index on Survival of Patients With Surgically Treated Renal Cell Carcinoma. J. Urol..

[8] Hakimi A.A., Furberg H., Zabor E.C., Jacobsen A., Schultz N., Ciriello G., Mikklineni N., Fiegoli B., Kim P.H., Voss M.H. (2013). An Epidemiologic and Genomic Investigation Into the Obesity Paradox in Renal Cell Carcinoma. JNCI J. Natl. Cancer Inst..

[9] Steffens S., Grünwald V., Ringe K.I., Seidel C., Eggers H., Schrader M., Wacker F., Kuczyk M.A., Schrader A.J. (2011). Does Obesity Influence the Prognosis of Metastatic Renal Cell Carcinoma in Patients Treated with Vascular Endothelial Growth Factor–Targeted Therapy?. Oncologist.

[10] Kalantar-Zadeh K., Horwich T.B., Oreopoulos A., Kovesdy C.P., Younessi H., Anker S.D., Morley J.E. (2007). Risk factor paradox in wasting diseases. Curr. Opin. Clin. Nutr. Metab. Care..

[11] Gu W., Wu J., Liu X., Zhang H., Shi G., Zhu Y., Ye D. (2017). Early skeletal muscle loss during target therapy is a prognostic biomarker in metastatic renal cell carcinoma patients. Sci. Rep..

[12] Harimoto N., Shirabe K., Yamashita Y.I., Ikegami T., Yoshizumi T., Soejima Y., Ikeda T., Maehara Y., Nishie A., Yamanaka T. (2013). Sarcopenia as a predictor of prognosis in patients following hepatectomy for hepatocellular carcinoma. Br. J. Surg..

[13] Miyamoto Y., Baba Y., Sakamoto Y., Ohuchi M., Tokunaga R., Kurashige J., Hiyoshi Y., Iwagami S., Yoshida N., Yoshida M. (2015). Sarcopenia is a Negative Prognostic Factor After Curative Resection of Colorectal Cancer. Ann. Surg. Oncol..

[14] Antoun S., Baracos V.E., Birdsell L., Escudier B., Sawyer M.B. (2010). Low body mass index and sarcopenia associated with dose-limiting toxicity of sorafenib in patients with renal cell carcinoma. Ann. Oncol..

[15] Prado C.M., Lieffers J.R., McCargar L.J., Reiman T., Sawyer M.B., Martin L., Baracos V.E. (2008). Prevalence and clinical implications of sarcopenic obesity in patients with solid tumours of the respiratory and gastrointestinal tracts: A population-based study. Lancet Oncol..

[16] Gu W., Zhu Y., Wang H., Zhang H., Shi G., Liu X., Ye D. (2015). Prognostic Value of Components of Body Composition in Patients Treated with Targeted Therapy for Advanced Renal Cell Carcinoma: A Retrospective Case Series. PLoS ONE.

[17] Ladoire S., Bonnetain F., Gauthier M., Zanetta S., Petit J.M., Guiu S., Kermarrec I., Mourey E., Michel F., Krause D. (2011). Visceral Fat Area as a New Independent Predictive Factor of Survival in Patients with Metastatic Renal Cell Carcinoma Treated with Antiangiogenic Agents. Oncologist.

[18] Antoun S., Lanoy E., Iacovelli R., Albiges-Sauvin L., Loriot Y., Merad-Taoufik M., Fizazi K., di Palma M., Baracos V.E., Escudier B. (2013). Skeletal muscle density predicts prognosis in patients with metastatic renal cell carcinoma treated with targeted therapies. Cancer.

[19] Antoun S., Birdsell L., Sawyer M.B., Venner P., Escudier B., Baracos V.E. (2010). Association of Skeletal Muscle Wasting With Treatment With Sorafenib in Patients With Advanced Renal Cell Carcinoma: Results From a Placebo-Controlled Study. J. Clin. Oncol..

[20] Powles T., Albiges L., Staehler M., Bensalah K., Dabestani S., Giles R.H., Hofmann F., Hora M., Kuczyk M.A., Lam T.B. (2018). Updated European Association of Urology Guidelines: Recommendations for the Treatment of First-line Metastatic Clear Cell Renal Cancer. Eur. Urol..

[21] Motzer R.J., Penkov K., Haanen J., Rini B., Albiges L., Campbell M.T., Venugopal B., Kollmannsberger C., Negrier S., Uemura M. (2019). Avelumab plus Axitinib versus Sunitinib for Advanced Renal-Cell Carcinoma. N. Engl. J. Med..

[22] Yip S.M., Heng D.Y.C., Tang P.A. (2016). Review of the Interaction Between Body Composition and Clinical Outcomes in Metastatic Renal Cell Cancer Treated With Targeted Therapies. J. Kidney Cancer VHL.

[23] McManus H.D., Zhang D., Schwartz F.R., Wu Y., Infield J., Ho E., Armstrong A.J., George D.J., Kruse D., Gupta R.T. (2023). Relationship Between Pretreatment Body Composition and Clinical Outcomes in Patients With Metastatic Renal Cell Carcinoma Receiving First-Line Ipilimumab Plus Nivolumab. Clin. Genitourin. Cancer.

